# Humanized Mouse Models of *Staphylococcus aureus* Infection

**DOI:** 10.3389/fimmu.2017.00512

**Published:** 2017-05-04

**Authors:** Dane Parker

**Affiliations:** ^1^Department of Pediatrics, Columbia University, New York, NY, USA

**Keywords:** *Staphylococcus aureus*, humanized mouse, pneumonia, lung, sepsis, skin, mouse model, infection

## Abstract

*Staphylococcus aureus* is a successful human pathogen that has adapted itself in response to selection pressure by the human immune system. A commensal of the human skin and nose, it is a leading cause of several conditions: skin and soft tissue infection, pneumonia, septicemia, peritonitis, bacteremia, and endocarditis. Mice have been used extensively in all these conditions to identify virulence factors and host components important for pathogenesis. Although significant effort has gone toward development of an anti-staphylococcal vaccine, antibodies have proven ineffective in preventing infection in humans after successful studies in mice. These results have raised questions as to the utility of mice to predict patient outcome and suggest that humanized mice might prove useful in modeling infection. The development of humanized mouse models of *S. aureus* infection will allow us to assess the contribution of several human-specific virulence factors, in addition to exploring components of the human immune system in protection against *S. aureus* infection. Their use is discussed in light of several recently reported studies.

## *Staphylococcus aureus* 

*Staphylococcus aureus* is a Gram-positive pathogen that can exist as a commensal on skin. It is a human pathogen and a leading cause of skin and soft tissue infections, pneumonia, endocarditis, and osteomyelitis (1, 2). In particular, methicillin-resistant *S. aureus* (MRSA) is a major problem not only in the hospital setting but also in the community causing significant economic burden (3–5). MRSA strains are twice as likely to kill and cost the US economy in excess of $4 billion/year (6–8). In contrast to hospital-acquired strains, community-acquired strains of *S. aureus* infect otherwise healthy individuals. The MRSA strain USA300 (4, 9, 10) infects healthy, hospitalized, and post-influenza patients in the context of pneumonia (11–14), is the dominant clone, and is epidemic in the United States. Secondary bacterial infection post-influenza is a leading cause of morbidity and mortality (15–17), which has been shown for history’s major pandemics, and *S. aureus* is one of the most common pathogens (12, 18, 19). This is of increasing concern as the population ages, as they are at increased risk of influenza infection. Colonization of the nose with *S. aureus* is relatively common with up to 30% of the population being persistent carriers, while the proportion colonized with MRSA is increasing (20–23). Carriage increases the risk of infection (24, 25), and as a result of this, patients are often decolonized prior to surgery to prevent infection (26).

## Mouse Models of Infection

Studies investigating the pathogenesis of *S. aureus* infection have relied heavily on the use of mouse models. Mice have been used to understand the role virulence factors play during infection as well as the contribution of specific host pathways and factors in the response to *S. aureus*. Mouse models for several important clinical diseases have been developed, including: peritonitis (27, 28), pneumonia (29–31), sepsis (32), skin and soft tissue infection (33, 34), endocarditis (35, 36), abscesses (37, 38), osteomyelitis (39, 40), arthritis (41), and nasal colonization (42–44).

Mice possess a number of attributes that make them desirable in modeling infection. They are small in size, do not occupy significant space, are cheap, reproduce rapidly, and have similar immune, nervous, cardiovascular, and endocrine systems to humans (45–47). Another major advantage is their genetic tractability. In mice, genes can be readily inactivated “knocked out,” genes inserted “knocked in,” gene reporter fusions integrated into the genome, and tissue specific mutations developed. This genetic utility makes them attractive to study host immune factors important in infection. However, the use of mice is not without their limitations. Many features of mice are significantly different from humans, such as their small size, altered metabolic rate, fatty acid composition of cells, higher rates of reactive oxygen species generation and thus oxidative damage, different diet, microbiome, and typically being inbred (48). There has also been some controversy recently on how well mice correlate with human inflammatory stresses based on transcriptional profiling and pathway analyses (49–51).

## Why Do We Need Humanized Mice for *S. aureus* Infection?

Although mice have proven extremely useful in determining the role of many *S. aureus* virulence factors and identifying host pathways that contribute to infection, they have been unable to predict success for vaccine candidates in humans (52, 53). This disconnect between the mouse model and efficacy in humans supports the conclusion that the mouse lacks all the necessary components to truly model *S. aureus* infection. It has also become increasingly apparent that *S. aureus* produces a number of virulence factors that have high species specificity toward the human molecular counterpart that they target.

One major group of proteins that possess human specificity are the bi-component toxins (54). Panton–Valentine leukocidin (PVL; LukSF), LukAB, and HlgCB, all preferentially target the human version of their receptor. PVL and HlgCB target the C5aR receptor, while LukAB targets CD11b (55). PVL does have some activity toward the rabbit version of the receptor; however, the other two toxins display only high specificity toward the human equivalent. The *S. aureus* superantigens/enterotoxins also show much greater affinity toward human cells, with vastly higher doses of protein required to invoke a response in mice (56, 57). *S. aureus* produces a large array of surface proteins required for its adherence to proteins encountered on the mucosal surface. Some of these surface proteins also display specificity toward their human counterpart, such as SdrG for human fibrinogen, Fnbp for fibronectin, and IsdB for hemoglobin (58). There are also likely to be several other yet-to-be-identified proteins that have human specificity based on the fact that *S. aureus* is a human-adapted pathogen. Thus, the development of a model that actually possesses the correct receptor targets and cells for these virulence factors to be investigated would be advantageous. The presence of an immune system to better model the human immune response would also no doubt prove useful in future vaccine development as well as gaining an improved understanding of the host–pathogen interaction in the context of *S. aureus* infection.

The host specificity of *S. aureus* toward human proteins has already been investigated in the context of superantigens and iron acquisition. It has been observed with the staphylococcal superantigens that HLA class II molecules control the superantigenic response and that this response is significantly reduced in non-human (including mice) models. A trend in this field has been to utilize knock-in mice expressing the appropriate HLA molecule for the superantigen (enterotoxin) under study. This has included HLA-DR3, HLA-DR4, and CD4 knock-in mice (59–63). Studies conducted using these mice have shown a significant increase in the immune response, indicative of the increased sensitivity of these cells to the superantigens. The preference for human hemoglobin over other mammal’s hemoglobin has been observed and is dependent upon the staphylococcal hemoglobin receptor IsdB. *S. aureus* grows better in the presence of human hemoglobin when iron is limited and the expression of human hemoglobin in mice leads to increased susceptibility to *S. aureus* infection (64). Thus, evidence already exists that warrants humanizing mice would improve the capacity to model *S. aureus* infection.

## Humanized Mice

The use of humanized mice has only relatively recently become prevalent. Their use was accelerated through the development of the NSG mouse (non-obese diabetic/severe combined immunodeficient mouse with a null mutation in the IL2R common gamma chain) (65). These mice lack B, T, and NK cells, complement, and have defective myeloid cells (65, 66). The NSG mice have been observed to possess the most efficient engraftment rates and support human hemato-lymphopoiesis (66–68). The mice are typically generated through the transfer of human CD34^+^ stem cells (69). Additionally, the implantation of human fetal liver/thymus tissue under the kidney capsule improves T cell development (70, 71). Humanized mice have been shown to evoke a human immune response to infection. The combinatorial diversity on their T cell receptors and IgG fully replicates the human samples that are used to populate the mice (72). Humanized mice have been utilized in the study of several viral pathogens such as EBV, HIV, and Dengue, as well as Malaria and *Salmonella* (73–76). Recently, a succession of studies has investigated the utility of these mice in the study of *S. aureus* pathogenesis.

## Recent Developments with *S. aureus* and Humanized Mice

The first study to investigate the utility of humanized mice with *S. aureus* highlighted their increased susceptibility to infection. Knop et al. (77) conducted intraperitoneal infections in humanized mice generated from irradiated NSG pups transferred with CD34^+^ cells. Humanized mice displayed significantly increased mortality compared to their controls. While non-reconstituted NSG mice did display some residual toxicity from radiation, the addition of human cells was shown to confer the lethality seen with the humanized mice. Increased bacterial counts were also observed in several organs; lungs, spleen, kidneys, liver, brain, and the bone marrow. The T cells in the humanized mice showed evidence of activation (CD69 expression), Fas receptor expression, and increased apoptosis after infection. Analysis of the human cells indicated a large proportion of B cells, followed by T cells and myeloid cells. Levels of chimerism were highest in the spleen (60%) and bone marrow (50%), 30% in the peripheral blood and <20% in the peritoneal exudate. This study indicated that humanized mice could be useful in modeling *S. aureus* infection, and subsequent studies have built on this to investigate the role of human-specific virulence factors.

The second study to utilize humanized mice with *S. aureus* investigated their utility in the context of skin infection, also showing an increased susceptibility to infection (78). In a subcutaneous model of infection, 10- to 100-fold less organisms were required to cause analogous disease pathology in non-humanized mice. Tseng et al. (78) found no differences in bacterial clearance or cytokine production. The phenotype observed was pathological, indicating that cellular toxicity did not influence bacterial clearance. The size of the skin lesions also correlated to the levels of chimerism in the mice, larger lesions were observed in mice with a higher percentage of human CD45^+^ cells. This model was then used to investigate the role of PVL in infection. PVL has a controversial role in infection. Conflicting epidemiological reports and animal studies exist, partly due to the fact many animal studies were performed prior to the identification of its receptor, C5aR, and its high preference for the human version of this receptor (79–87). The expression of PVL led to larger areas of dermonecrosis. This effect was due to its ability to target and kill neutrophils, as transfer of human neutrophils alone to NSG mice was able to recapitulate this phenotype. While the authors successfully showed a role for PVL in skin infection with molecular Koch’s postulates, a PVL inhibitor *in vivo* was unable to reduce disease severity. Like the first study, this work also utilized stem cell transfer into neonate NSG mice and observed similar levels of engraftment in the spleen. This work proved the utility for the humanized mouse in delineating the functions of staphylococcal virulence factors as well as its usefulness as a model for skin infection.

The third and most recent humanized mouse study showcased the utility of these mice for respiratory infection (71). As in the previous studies, the humanized mice displayed a significant increase in susceptibility to infection. Compared to the standard mouse strains C57BL/6J, NOD and murinized controls (NSG mice transferred with murine bone marrow), the humanized mice contained bacterial burdens 40-fold higher. The role of PVL was also investigated in this pulmonary model and was shown to contribute to infection, using both bacterial mutants and neutralizing antibody (71). The presence of PVL led to increased bacterial burden, increased lung pathology and decreased cytokine production. The target of PVL appeared to be the macrophage, with increased numbers present in mice infected with the PVL-deficient strain. The NSG transgenic mouse with human *Il3* and *Csf2* knocked in has improved macrophage reconstitution compared to the standard NSG humanized mouse (88). Consistent with human macrophages conferring the increased susceptibility, the use of these additional knock-in mice had even higher levels of bacteria present in the airways and lung tissue. While a role for PVL in pulmonary infection was identified, this was not the case for another human-specific toxin LukAB, which displayed no phenotype in this model (71). This study differed from the previous two in its use of adult mice and the implantation of thymus tissue under the kidney capsule. This was apparent in the higher levels of T cells present among the human CD45^+^ population, approximately 50% in the lung (71). What these three studies do show is that irrespective of the inoculation site the humanized mice had an increased susceptibility to infection, which will only improve as better humanized mouse models are generated.

## Future Models

The development of improved humanized mouse models will further increase the susceptibility and hence sensitivity of modeling *S. aureus* infections *in vivo*. This will be achieved through improved overall reconstitution of the human immune system, improved differentiation, and development of myeloid subsets, as well at the improved expression of neutrophils, an integral cell type particularly in pneumonia and skin infection models. Significant work has already been done in this area with the insertion of *Csf1, Csf2*, and *Il3* into mice, leading to improved differentiation of macrophages and alveolar macrophages, respectively (88, 89). The knocking in of *Csf2* and *Il3* was shown to increase the susceptibility of *S. aureus* in the context of acute pneumonia (71). Further studies have shown that the integration of thrombopoietin enhances maintenance and multilineage differentiation and insertion of signal-regulatory protein alpha prevents phagocytosis of the human cells by the remnant murine immune system (90, 91). Additional transgenics appropriate to *S. aureus* would include a combination of the aforementioned along with: human HLA types for the study of superantigens (92), insertion of human toll-like receptors for the innate immune response (75), as well as the incorporation of epithelial cells in the lung and skin for mucosal models (34, 93) and red blood cells for systemic studies (94, 95). These developments will facilitate adequate modeling of a broad range of *S. aureus* human-specific virulence factors.

## Conclusion

*Staphylococcus aureus* is a significant human pathogen that has long been modeled in mice. Studies to-date in mice have delineated the roles of various bacterial and host factors important in infection; however, data on potential vaccine candidates identified in these models have not had similar success in human studies. Recent studies utilizing humanized mice have illuminated their utility in models of peritonitis, skin and soft tissue infection, and pneumonia. Researchers have shown humanized mice have increased susceptibility to *S. aureus* and in skin and pneumonia models a role for PVL in infection has been identified. As the next generation of humanized mouse models are developed, the capacity for modeling *S. aureus* will only improve. Humanized mice will facilitate determining the role of virulence factors with human host specificity and hopefully provide a system whereby potential vaccine candidate translate efficacy to humans.

## Author Contributions

DP conceived and wrote the manuscript.

## Conflict of Interest Statement

The author declares that the research was conducted in the absence of any commercial or financial relationships that could be construed as a potential conflict of interest.
